# Effects of Smoking on Aggression, Big Five Personality Factors, and Polymorphisms in HTR2A, DRD4, and MAOA among Egypt University Students

**DOI:** 10.1155/2022/1879270

**Published:** 2022-09-13

**Authors:** Marina Aboelsaad, Omar Soliman, Ahmed Medhat, Omar Khalil, Mawada AlWahsh, Yasmeen Wageh, Amira ElSaied, Hadeer ElShrkawy, Huda Abdulhafiz, Moustafa Sayed

**Affiliations:** ^1^Clinical Pharmacy Practice Department, Faculty of Pharmacy, The British University in Egypt, Egypt; ^2^Centre of Drug Research and Development, Faculty of Pharmacy, The British University in Egypt, Egypt

## Abstract

**Introduction:**

To find genetic variants in the DRD4 and HTR2A genes' promoter regions and exons that are associated with tobacco smoking and nicotine addiction in Egyptian university students as well as to study the association between personality traits and smoking status.

**Methods:**

A self-administered questionnaire about cigarette smoking and personality attributes (Big Five Inventory, ESPAD Questionnaire on Substance Abuse, and Buss-Perry Aggression Questionnaire). The participants in the study were 90 nonsmokers (NS) and 88 current smokers (CS), who were divided into two groups depending on their cigarette consumption per day (cpd): 55 heavy smokers (HS, >20 cpd) and 33 light smokers (LS, 1–10 cpd). Four and eight single nucleotide polymorphisms (SNPs) in the DRD4, HTR2A, and MOA genes, respectively, were genotyped.

**Results:**

Smokers scored lower on neuroticism, agreeableness, conscientiousness, openness, and extraversion than nonsmokers, but higher on aggression. Furthermore, the C allele of rs1800955 in DRD4 was associated with cigarette smoking in the HS vs. NS and LS vs. NS studies. The T allele of the HTR2A rs6313 gene was discovered to be strongly associated with cigarette smoking. There was no link discovered between MOA rs1137070 and MOA rs1137070.

**Conclusions:**

Using a comprehensive personality model (FFM), this study repeats and extends earlier research. Personality and genetic studies may aid in the development of a more complete and conclusive understanding of cigarette smoking, as well as more precise policies and guidelines for smoking cessation and quitting.

## 1. Introduction

There are approximately 1.3 billion cigarette smokers worldwide [1], where in the United States, there are around 34.1 million adults who currently smoke cigarettes [2]. Cigarette smoking continues to be a major cause of preventable disease, disability, and mortality in the United States, accounting for about 480,000 deaths annually or almost one in every five deaths [3].

To improve the effectiveness of smoking-based prevention and therapy, programs are continuously developed to tailor interventions to individual risk factors and social risk factors of smoking (e.g., incentive to change and psychiatric background) [4, 5]. Personality traits, which reflect individual variation in enduring psychological traits [6–8], have received a great deal of attention in terms of their effect on smoking behavior [8–13]. Furthermore, personality traits like neuroticism are frequently associated with cigarette smoking [14–16]. Even though previous research looked at a variety of personality definitions and models, the Five-Factor Model (FFM) has been an increasingly well-accepted model of personality dimensions with high influence [17–19]. The FFM hypothesizes that personality traits encompass five higher-order dimensions, including openness to new experience (innovation, risk-taking, and openness to new ideas), conscientiousness (organization and discipline), extraversion (assertiveness, sociability) neuroticism (predisposition to experience negative emotions), and agreeableness (the extent to which behavior is typically seen as compliant and cooperative) [19, 20]. These five higher-order characteristics are heritable, extremely stable across time, and noticeable in diverse social settings [19].

Several studies have demonstrated a link between the Big Five personality traits and smoking habits. Those who are high in extraversion smoke to seek excitement, whereas those who are high in neuroticism smoke to relieve stress and anxiety, according to Eysenck [21]. Low conscientiousness is linked to harmful health habits, such as smoking, according to research on health risk behaviors. The Situation-Trait-Adaptive-Response model of smoking was suggested, demonstrating that neuroticism, extraversion, and psychoticism (along with low conscientiousness and low agreeableness, which are now recognized traits) were all positively related with smoking [9, 22–25]. Recent research, however, has found that extraversion, which is often viewed as a socially beneficial trait, had little or no effect, possibly due to changes in cultural acceptance of smoking through time. Following that, a meta-analysis of the Big Five and smoking was undertaken, and smokers were found to have higher neuroticism, as well as poorer agreeableness and conscientiousness [22, 26]. Even though Hakulinen et al. found a significant influence of extraversion personality characteristic on smoking in another meta-analysis, the effect size was quite minor [27]. Moreover, in recent studies in one of two samples analyzed by Sallis et al. [28], extraversion had a strong yet little influence. Graham et al. also did an integrative data analysis of 16 worldwide longitudinal studies, which yielded comparable results [29]. This study was designed to find the prevalence and correlation between smoking, aggression, personality traits, and genetic variants in the promoter regions and exons of the *DRD4*, *HTR2A*, and *MAOA* genes among university students in Egypt.

## 2. Materials and Methods

Study participants were recruited from different Egyptian universities, in which their age was within 19–24 years old. Level of smoking was categorized as follows: nonsmoker, light smokers (individuals who smoked 1-10 cigarettes/day), and heavy smokers (individuals who smoked 10 or more cigarettes/day). Moreover, different questionnaires were used to measure personality traits, aggression, lifetime use, and frequency of smoking of the participants in which all were self-reported as follows.

### 2.1. Big Five Inventory (BFI)

A 50-item questionnaire was used to measure individual personality traits: openness (innovation, risk-taking, and openness to new ideas), extraversion (assertiveness, sociability), neuroticism (predisposition to experience negative emotions), conscientiousness (organization and discipline), and agreeableness (the extent to which behavior is typically seen as compliant and cooperative) [30].

### 2.2. ESPAD Questionnaire on Substance Abuse

To measure the frequency and lifetime use of cigarettes, only the questions about cigarette smoking (4 questions) were used for obtaining results relevant to cigarette smoking in our study [31].

### 2.3. Buss-Perry Aggression Questionnaire

A 29-item questionnaire developed by Puss and Perry which classified aggression into four factors, physical aggression, verbal aggression, hostility, and anger (Buss & Perry [32]), was used to correlate the degree of aggression with smoking behavior.

### 2.4. Genotyping

Furthermore, DNA samples were collected from participants using a sterile cotton swab to detect single nucleotide polymorphisms in dopamine receptor D4 particularly DRD4 rs1800955, serotonin receptor particularly HTR2A rs6313, and monoamine oxidase-A specifically MAOA rs1137070 using SNP arrays.

Three litres of DNA at a concentration of 15 ng/L were utilised for real-time PCR genotyping. The following were the amplification parameters: 50°C for 2 minutes, 95°C for 10 minutes, 40 cycles of 95°C for 15 seconds, and one minute at 60°C. Real-time PCR (7300 Real-Time PCR System, Applied Biosystems, Foster City, CA, USA) was used to define the alleles and genotypes of the polymorphisms using allelic discrimination using commercial TaqMan probes at a 40x concentration (Applied Biosystems, Foster City CA, USA). In addition, for each genotyping plate, three nontemplate controls (contamination controls) were included, and 1% of the samples in the research was genotyped in duplicate for control allele assignment.

### 2.5. Ethics Statements

The Ethical Committee of the Faculty of Pharmacy at the British University in Egypt examined and approved this study. The study's goal was explained to participants who were invited to participate. They then signed a letter of informed consent. With the goal of maintaining confidentiality, each participant was given an alphanumeric key.

### 2.6. Data Availability Statement

The datasets generated during and analyzed during the current study are available from the corresponding author on reasonable request.

### 2.7. Statistical Analysis

Using the statistical program SPSS v.15.0, the mean and standard deviation (SD) of each variable were calculated (SPSS software, IBM, New York, USA). The following comparisons were made to find the genetic markers linked to cigarette consumption: HS vs. NS and LS vs. NS. Bonferroni's test was used to adjust the significant values in each comparison.

## 3. Results

### 3.1. The Relationship of Tobacco Smoking and Personality Trait Score

Table 1 shows the means (SD) for personality attributes for the never, light, and heavy smoker groups. According to the analysis of variance, there were significant differences in aggression, openness, neuroticism, conscientiousness, agreeableness, and extraversion among the groups. Gender differences, on the other hand, did not appear to have an impact on smoking among the students who took part in the study.

### 3.2. Relationship between Different Genotypes of DRD4, HTR2A, and MAOA and Smoking Status

There was no significant difference in the genotype distribution of MAOs (rs1137070) among the 3 groups (*P* = 0.506) (Table 2). There was a significant difference in the TT and CT and CC genotype distribution of DRD4 (rs1800955) among the 3 groups (*P* = 0.029). The CC genotype of DRD4 rs1800955 only appeared in the LS and HS groups (16.7 and 25%, respectively). There was a significant difference in the TT and CT and CC genotype distribution of HTR2A rs6313 among the 3 groups (*P* = 0.012). The TT genotype of HTR2A rs6313 only appeared in the LS and HS groups (16.7 and 25%, respectively) (Table 2).

## 4. Discussion

This study assured the existence of a significant correlation between smoking and some personality traits (extraversion, neuroticism, conscientiousness, openness, agreeableness, and aggression). One of the important aspects of this study was that we used the Five-Factor Personality Model (FFM) to achieve the abovementioned outcome in Egypt university students. We show here that smokers scored significantly higher than nonsmokers on aggression and significantly lower on agreeableness, conscientiousness, openness, extraversion, and neuroticism.

In contrast to others who suggested high neuroticism as a risk factor smoking initiation, our data shows that smokers actually scored lower on neuroticism when compared to nonsmokers which could be attributed to a stimulus-response effect of smoking [33]. Furthermore, nicotine depletion, identified as period between smokes, is thought to cause strong negative emotional states [34]. However, this could be different from one society to another which was the case in our study.

Openness is reported to be associated with behavioral disinhibition, novelty seeking, and behavioral activation [35]. It is thought that those who are more open to new experiences are more eager to smoke tobacco while they are young, which can lead to addiction later on. We, on the other hand, have the contrary report.

Worldwide, extraversion is unrelated to the status of smoking. Prior findings which suggested that extraversion is related to smoking were based on the fact that impulsivity is one of the extraversion's facets included [36]. Extraversion is measured globally using a variety of questionnaires with various features, which explains the literature's contradictory findings on the broad extraversion component. Furthermore, extraverts are more prone to smoke in communities with a high frequency of smoking since smoking is less socially criticized [10]. Some studies reported a significant relationship between extraversion and cigarette smoking, [37, 38]. On the other hand, other studies reported the opposite [39, 40]. An inverse pattern reported by our study could be attributed to the fact that introverts may seek smoking to compensate for unsociability in the Egyptian society.

It is not surprising to report lower scores on agreeableness for smokers compared to nonsmokers. According to a few research, rebelliousness, a feature closely associated with poor agreeableness, is a primary driver of cigarette smoking aetiology [41]. Individuals with low agreeableness scores are aggressive, intolerant, and antagonistic; they have lower social approval demands and are thus more likely to start and continue smoking despite the harmful effects of smoking on others. Surprisingly, aggression is thought to be a smoking risk factor [42]. The current study replicates previous work [43–45], showing that smokers score higher on self-report aggression than nonsmokers. Thus, there is a perfect correlation between lower agreeableness and high aggression scores reported in this study.

Trobst et al. have reported that low conscientiousness is associated with other health risk behaviors [46], which supports the fact that there is a negative association between conscientiousness and smoking [47]. Given that this ability is linked to a healthy lifestyle, high conscientiousness reflects self-control and planning capacity. Our results confirm the negative relation between smoking and conscientiousness.

It is believed that a genetic component is associated with nicotine addiction, with several genetic variants being reported. The rs1800955, which is found in the promoter region (-521 C/T), is the most commonly studied SNP in the DRD4 genes. We found that only the C allele of rs1800955 is linked to cigarette smoking among Egyptian university students and that the link was maintained even in the homozygous CC genotype. Other research has suggested that having the T allele in rs1800955 reduces the number of dopamine D4 receptors at the synapse [48]. As a result, we suggest that smokers' brains with the C allele have more D4 dopamine receptors than nonsmokers' brains with the T allele, which may be the cause of smoking habit.

For the HTR2A gene, we have found that the T allele of rs6313 was associated with tobacco consumption. Gloria et al. have reported that the TT genotype of rs6313 in the Australian population was associated with a higher risk of cigarette smoking [49]. These results are matched with the ones reported in our study. In addition, Polesskaya et al. reported that the presence of the C allele in rs6313 reduced the expression of the HTR2A gene by 20% compared with the T allele [50]. Functional studies are required to test this hypothesis. Currently, our study allows us to conclude that the C allele of rs1800955 in the DRD4 gene and the T allele of rs6311 in the HTR2A gene are associated with cigarette smoking. In the current study, we investigated the interactions between MAOA rs1137070 and smoking pattern; however, there was no significant correlation.

## 5. Study Limitations

We acknowledge that this study has some limitations. First, this was a cross-sectional study which cannot help determine the impact of different variables. In addition, self-reported questionnaires may be biased especially in tobacco consumption. Third, the students who participated in this study were limited to the university undergraduate students, which could restrict the generalization of results to other age groups. Furthermore, SNPs in genes associated with nicotine metabolism, such as CYP2A6 and CYP2B6, were not taken into account.

## Figures and Tables

**Table 1:** The relationship of tobacco smoking and personality trait score. It shows the means (SD) for personality attributes for the non-, light, and heavy smoker groups.

Characteristics	Cigarette smoking status			*P*
	NS	LS	HS	
Gender				
Male	34 (32.4)	21 (20)	50 (47.6)	>0.05
Female	46 (73)	12 (19.1)	5 (7.9)	
Aggression score	74.39 ± 25.03	80.13 ± 24.77	88.3 ± 25.69	<0.001
Openness score	24.49 ± 6.34	23.91 ± 5.13	21.71 ± 7.09	<0.001
Neuroticism score	19.06 ± 8.17	18.25 ± 6.65	16.93 ± 6.59	<0.001
Conscientiousness score	23.31 ± 5.99	22.17 ± 5.98	20.63 ± 5.48	<0.001
Agreeableness score	25.88 ± 7.16	23.78 ± 5.37	23.97 ± 5.27	<0.001
Extraversion score	19.59 ± 6.78	18.21 ± 6.36	19.08 ± 5.48	<0.001

Analysis of variance indicates that there were significant differences among groups on aggression, openness, neuroticism, conscientiousness, agreeableness, and extraversion factors.

**Table 2:** Genotypes in the 3 groups.

Genotypes	NS (*N* = 33)	LS (*N* = 12)	HS (*N* = 32)	*P*
DRD4 rs1800955				
TT	51.5	41.67	51	0.029
TC	42.5	33.33	31	
CC	6	25	18	
HTR2A rs6313				
CC	66.6	66.66	62.5	0.012
CT	33.33	16.66	12.5	
TT	0	16.66	25	
MAOA rs1137070				
CC	29.1	58.3	37.5	0.506
CT	34.37	25	25	
TT	36.36	16.7	37.5	

Data expressed as percentage. NS: nonsmokers; LS: light smokers; HS: heavy smokers.

## Data Availability

The data for this project are confidential, but may be obtained with Data Use Agreements with the British University in Egypt. Researchers interested in access to the data may contact Dr. Moustafa Sayed at helmy.mostafa@bue.edu.eg.

## Additional Points

*Implication Section*. This study provides new insights about personality traits and smoking addiction in Egypt youth. It also gives for the first time in Egypt some data about associated genes to smoking habits.
